# Droplet Control Based on Pinning and Substrate Wettability

**DOI:** 10.1021/acs.langmuir.1c00215

**Published:** 2021-04-05

**Authors:** Panagiotis E. Theodorakis, Alidad Amirfazli, Bin Hu, Zhizhao Che

**Affiliations:** † Institute of Physics, Polish Academy of Sciences, Al. Lotników 32/46, 02-668 Warsaw, Poland; ‡ Department of Mechanical Engineering, York University, Toronto, M3J 1P3 Ontario, Canada; § Flow Capture AS, Industriveien 1, 2020 Skedsmokorset, Norway; ∥ State Key Laboratory of Engines, Tianjin University, 300072 Tianjin, China

## Abstract

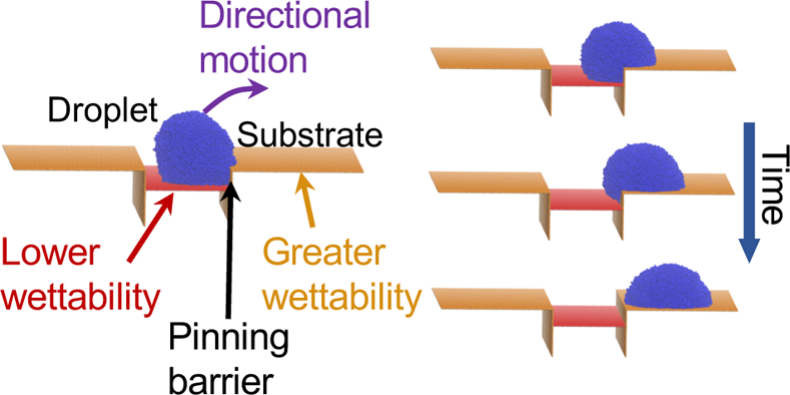

Pinning of liquid droplets on solid substrates is ubiquitous and plays an essential role in many applications, especially in various areas such as microfluidics and biology. Although pinning can often reduce the efficiency of various applications, a deeper understanding of this phenomenon can actually offer possibilities for technological exploitation. Here, by means of molecular dynamics simulation, we identify the conditions that lead to droplet pinning or depinning and discuss the effects of key parameters in detail, such as the height of the physical pinning barrier and the wettability of the substrates. Moreover, we describe the mechanism of barrier crossing by the droplet upon depinning, identify the driving force of this process, and, also, elucidate the dynamics of the droplet. Not only does our work provide a detailed description of the pinning and depinning processes but also it explicitly highlights how both processes can be exploited in nanotechnology applications to control the droplet motion. Hence, we anticipate that our study will have significant implications for the nanoscale design of substrates in micro- and nanoscale systems and will assist with assessing pinning effects in various applications.

## Introduction

The control of droplets on solid substrates is crucial for many applications in various areas, such as microfluidics, microfabrication, coatings, and biology. To this end, the accurate steering of droplet motion can be realized by a proper substrate design. In materials science, for example, a design based on micropillar structures has been shown to lead to superhydrophobic substrates^1^ for, among others, self-cleaning^2^ and anti-icing.^3^ As a result of this specific design, pinning effects naturally arise that may affect a droplet’s motion by introducing a sticky or slippery behavior,^4−8^ which also depends on the substrate wettability.^9,10^ By means of the lubrication theory, Joanny and Robbins have investigated the dynamics of a contact line on a heterogeneous plate, which is advanced at a constant force or velocity.^11^ They have unveiled the scaling of the force and the velocity and, also, found that alternating patches of constant wettability produce a linear relation. Espín and Kumar have presented a model based on the lubrication theory to describe contact line pinning on substrates with heterogeneities. The work has discussed the effect of roughness through a continuum model that has shown to agree with experiments.^12^ Alava and Dubé have analyzed the statistical properties of the spreading contact line (droplet radius and contact angle) on heterogeneous surfaces.^13^ Moreover, Marmur has described the equilibrium wetting on rough surfaces determining the transition between homogeneous and heterogeneous wetting regimes on the basis of the Wenzel and Cassie–Baxter equations.^14^ Experimentally, Ramos and Tanguy have studied the pinning–depinning phenomena of a contact line on a solid surface decorated by a random array of nanometric structures and found a linear relation between the hysteresis caused by defects and their areal density.^15^ In this context, the relation between the dynamic contact angle and the contact line speed has been recently considered by numerical simulations.^16^ In another example, substrates characterized by a gradient of a physical or chemical property in a particular direction along the substrate can steer the motion of liquid droplets without the requirement of an external energy source.^17−21^ A well-known example is durotaxis, where a droplet can autonomously move along a substrate due to the presence of a stiffness gradient,^22−25^ which crucially depends on the wettability of the substrate.^24^ In any of the above systems, pinning of the contact line can be advantageous or impede the droplet motion or its manipulation, leading to greater or lower efficiency of relevant processes.^4,5,26−31^ There are still outstanding issues that remain regarding the possibility of exploiting the effects of droplet pinning and substrate wettability in controlling droplet motion. This is especially true regarding microlevel origins of pinning and its mechanism, which can be advantageous for various nanotechnology applications.

**Figure 1:**
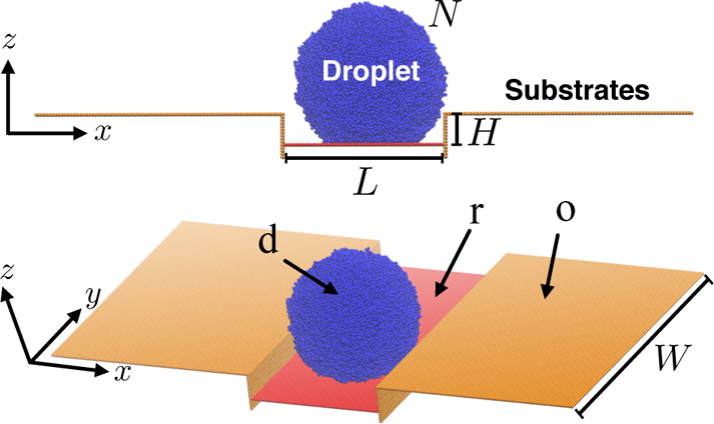
Typical initial configuration of our simulations. Two different views of the same configuration are presented in the upper and the lower panels for the sake of clarity. The system consists of substrates with different wettabilities indicated by different colors [red (r) and orange (o)]. The droplet (d) consists of blue beads and is placed onto the red substrate. The pinning barrier is characterized by the height, *H*, between the parallel (along the *x* – *y* plane) red and orange substrates. Different values of *H* and different wettabilities for the red and orange substrates are considered in this study. The length, *L*, depends on the size of the droplet and the wettability of the red substrate and is large enough to guarantee that the droplet is in a state as the one illustrated in the upper panel with an appropriate distance between the droplet and the lateral orange substrate (perpendicular to the *x* – *y* plane). The width *W* is chosen to guarantee that mirror images of the droplet in the *y* direction are not interacting due to the periodic boundary conditions, which are applied in all directions. Here, the example refers to a droplet with *N* = 50,400 coarse-grained beads, *H* = 12σ, and the interaction between the droplet and the red substrate ε_rd_ = 0.3ε. Snapshots have been produced using VMD software.^33^

This paper aims at filling the above gap by taking advantage of high-fidelity *in silico* experiments on the nanoscale. We employ molecular dynamics (MD) simulation based on a coarse-grained model and the system setup of Figure 1. Apart from aiming at acquiring an in-depth understanding of droplet pinning on solid substrates with different wettability, we also argue that the pinning has the potential of controlling nanodroplets, for example, selective droplet separation. For this reason, we have studied a range of different pinning scenarios, which include various combinations of substrate wettabilities and pinning barriers for droplets of different sizes. Thus, we anticipate that our results will inspire the design of substrates for steering droplets in micro- and nanoscale systems and will assist with assessing pinning effects in a range of different nanotechnological applications.

## Materials and Methods

We have used MD simulations of a coarse-grained model^24,32^ where interactions between different components of the system, that is, the drop and the substrate beads, are described by means of the Lennard-Jones (LJ) potential, namely
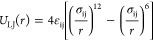
1where *r* is the distance between any pair of beads in the system, and i and j indicate the type of beads: “d” for droplet beads, “r” for the beads that belong to the red substrate, and “o” for the beads of the orange substrates (Figure 1). In our model, σ_ij_ = σ for all combinations of types i and j, with σ being the unit of length. As usual, the LJ potential is cut and shifted at a cutoff distance *r*_c_ = 2.5σ for any interaction involving the droplet beads, while *r*_c_ = 2^1/6^σ (purely repulsive potential) for any interactions between the substrate beads. The strength of the interactions is defined by the parameter ε_ij_ of the LJ potential. In our case, the parameters ε_rd_ and ε_od_ vary between 0.3ε and 0.7ε, where ε is the energy unit and *k*_B_ (Boltzmann’s constant) is considered as unity.^24^ The interactions ε_rd_ and ε_od_ are used to tune the wettability of the droplet on the red and the orange substrates (Figure 1).

We have considered droplets of different sizes, which consist of *N* = 112, 1008, or 5040 chains of 10 coarse-grained beads each. The finite extensible nonlinear elastic (FENE) potential^32^ was used to tether together consecutive beads in these polymer chains, which is mathematically expressed as follows
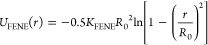
2where *r* is the distance between two consecutive beads along the polymer backbone, *R*_0_ = 1.5σ expresses the maximum extension of the bond, and *K*_FENE_ = 30ε/σ^2^ is an elastic constant. For the chosen chain length, there are no evaporation effects and the vapor pressure is therefore sufficiently low.^24,34^

To evolve our system in time, we used MD simulation by choosing the Langevin thermostat^35^ as implemented in the LAMMPS package.^36^ The time unit in our simulations is , where *m* is the mass unit. The time-step for the integration of the equations of motion for the droplet particles is Δ*t* = 0.005τ. Thus, the temperature *T* fluctuates around a predefined value *T* = ε/*k*_B_, where *k*_B_ is the Boltzmann constant, and the energy ε is measured in units of *k*_B_*T*. Periodic boundary conditions are applied in all directions, and we guarantee that mirror images of the droplet do not interact with each other in any direction. A typical initial configuration for our systems is illustrated in Figure 1. Typical trajectories for our systems start from such initial configurations. We have run simulations up to 10^8^ MD time steps for cases that remained pinned to ensure that unpinning will not happen at a very late time of the simulation. For droplets that cross the pinning boundary, the length of the trajectories was up to the point that the droplet reached the final equilibrium state on top of the orange substrate. Our results are based on the analysis of these trajectories.

## Results and Discussion

Before delving into the details of the system, it should be mentioned that pinning can be the result of chemical inhomogeneity, surface roughness (or a physical step), or a combination of both. In this work, we will consider the combined effect of the physical barrier and wettability to allow for a comprehensive understanding. Pinning is defined as the inability of the contact line to move; such inability is rooted in the thermodynamic energy barrier due to chemical and/or physical heterogeneity expressed on a surface. In this study, such a barrier to the movement of the contact line is through the physical barrier that prevents the droplet from moving on top of the orange substrate; the wettability of the physical heterogeneity is also varied. Due to the attractive nature of the LJ interaction, the droplets in this study are pinned at the boundary between the red and orange substrates as such the pinning inherently takes place without imposing a pinning requirement. The system studied here consists of a droplet on a substrate that is parallel to the *x* – *y* plane, as shown in Figure 1. The wettability of the substrate by the droplet is determined by the LJ interaction parameter, ε_rd_, where “r” indicates the red color of the substrate and “d” the droplet (Figure 1). A larger value of ε_rd_ allows for higher wettability of the substrate, whereas a smaller value corresponds to lower wettability. From our previous study,^24^ the choice, 0.3ε ≤ ε_rd_ ≤ 0.7ε, maintains the spherical-cap shape of the droplet on a substrate monolayer and avoids the evaporation effects and large distortions of the droplet contact line. In this case, the contact angle of the droplet is uniquely defined by the strength of the LJ interaction (*e.g.*, ε_rd_) and linearly depends on it.^37^ In particular, LJ energy parameters in the range 0.3–0.7ε would yield contact angles in the range 60–120°.^24^ In addition, two orange substrates perpendicular to the *x* – *y* plane and two orange substrates parallel to the *x* – *y* plane are part of the same system as illustrated in Figure 1. Both orange substrates have the same wettability, which is expressed by the interaction strength of the LJ potential, ε_od_, where “o” stands for the orange color of the substrates. The orange substrates, which are parallel to the *x* – *y* plane, and the red substrate are separated by a distance, *H*, in the *z* direction, which corresponds to the height of the physical barrier that the droplet needs to overcome in order to move from the red substrate to the orange substrate. The pinning barrier, namely the height, *H*, can vary by changing the position of the red substrate in the *z* direction. The choice of lengths, *L* and *W* (Figure 1), does not affect our results. *L* is chosen such that the droplet sticks to the pinning barrier after a short time since the interaction of the droplet with the red and the orange substrates is always attractive. *W* is large enough to guarantee that mirror images of the droplet do not interact in the *y* direction due to the presence of the periodic boundary conditions. Hence, depending on the choice of the parameters, *H*, ε_rd_, and ε_od_, as well as the droplet size (total number of beads, *N*), the droplet may be able to overcome (cross) the pinning barrier and potentially reach a new equilibrium state on top of one of the orange substrates. In the following, we discuss the effects of these parameters on droplet pinning and describe the mechanism of droplet motion over the barrier upon droplet depinning.

**Figure 2:**
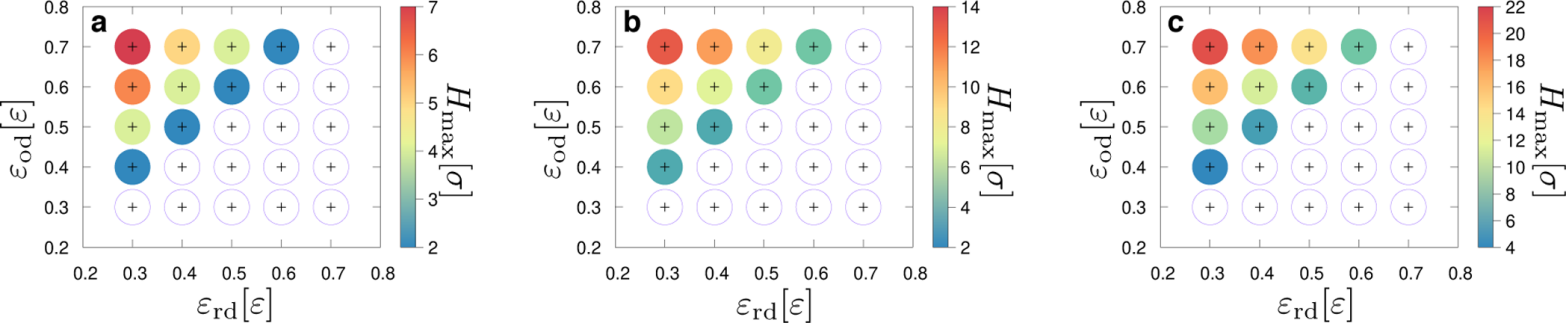
Maximum height of the pinning barrier, *H*_max_, that a droplet is able to overcome for different values of wettability for the red and orange substrates, as expressed *via* the parameters ε_rd_ and ε_od_, respectively. The black crosses indicate the exact values of ε_rd_ and ε_od_ on the graphs for each circle. The color code reflects the value of *H*_max_ for each set of parameters. The empty circles indicate that the droplet remains pinned (on the red substrate) for any value of *H* ≥ 1.0σ. Each plot refers to droplets of different sizes, namely, *N* = 1120 (a), *N* = 10,080 (b), and *N* = 50,400 (c) beads.

Figure 2 presents the results on the maximum height of the pinning barrier, *H*_max_, that the droplet is able to overcome. In particular, the dependence of *H*_max_ on the parameters ε_rd_ and ε_od_ for droplets of different sizes is laid out. We observe that the droplet will remain pinned, when the red substrate has a greater wettability than the orange substrates, independently of the droplet size. In other words, ε_od_ must always be larger than ε_rd_ to allow for droplet depinning. Hence, the thermal fluctuations of the droplet alone are not sufficient to enable depinning, even for values of *H* as low as *H* = σ and even for our largest droplets (*N* = 50,400 beads). However, droplets can generally overcome ever larger barriers as their size increases when ε_od_ > ε_rd_ and for the range of values considered in this study. In particular, *H*_max_ can be as high as 21σ in the case of a droplet consisting of *N* = 50,400 beads (Figure 2c, ε_rd_ = 0.3ε and ε_od_ = 0.7ε). In contrast, a droplet of *N* = 1120 beads would only overcome a barrier of 7σ at best (Figure 2a, ε_rd_ = 0.3ε and ε_od_ = 0.7ε). Moreover, the value of *H*_max_ crucially depends on the wettability difference between the red and the orange substrates in each case, as expressed through the LJ parameters ε_rd_ and ε_od_. In particular, the larger the difference in wettability, the larger the *H*_max_ the droplet is able to overcome. In other words, as the difference in wettability between the red and orange substrates becomes smaller, *H*_max_ decreases. In addition, choosing the highest possible wettability for the orange substrates always yields the largest *H*_max_, which suggests that maximizing ε_od_ favors droplet depinning. For example, the combination (ε_rd_ = 0.5ε, ε_od_ = 0.7ε) results in a larger value of *H*_max_ in comparison with the combination (ε_rd_ = 0.3ε, ε_od_ = 0.5ε) in the case of all droplet sizes, despite the absolute difference between the parameters ε_rd_ and ε_od_ being the same. Eventually, the affinity of the droplet to the orange substrates drives the crossing of the barrier, as will be discussed further below. In summary, the largest *H*_max_ is achieved for (ε_rd_ = 0.3ε, ε_od_ = 0.7ε) and the smaller *H*_max_ for the combination (ε_rd_ = 0.3ε, ε_od_ = 0.4ε). In view of these observations, we present in the following results of pinning and depinning (see also movies in the Supporting Information) by keeping ε_rd_ = 0.3ε constant and varying ε_od_ as well as results where we keep ε_od_ = 0.7ε constant, and vary ε_rd_.

**Figure 3:**
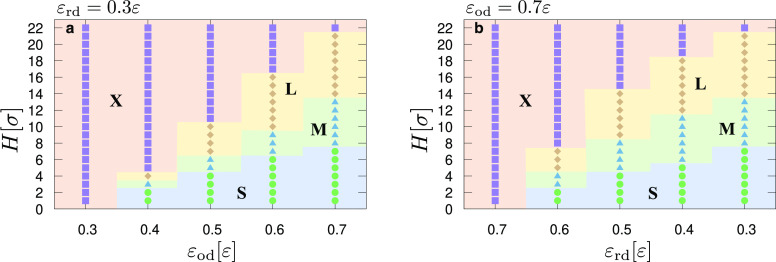
(a) State diagram (pinning/depinning) for droplets of different sizes (small-size droplets with *N* = 1120 beads, medium-size droplets with 10,080 beads, and large-size droplets with 50,400 beads) as a function of the height, *H* (vertical axis), and the interaction parameter ε_od_ (horizontal axis). ε_rd_ = 0.3ε. “S” indicates cases (circles) for which small, medium, and large droplets are able to overcome a barrier, *H*. “M” indicates cases (triangles) for which only the medium and large droplets are able to overcome a pinning barrier of height *H*. “L” indicates cases (diamonds) for which only the large droplets can overcome a pinning barrier of height *H*. Finally, “X” indicates cases (squares) for which the droplets remain pinned, irrespective of their size. (b) Similar to panel (a), but results refer to cases where ε_od_ = 0.7ε and ε_rd_ varies (horizontal axis), as indicated on the graph.

**Figure 4:**
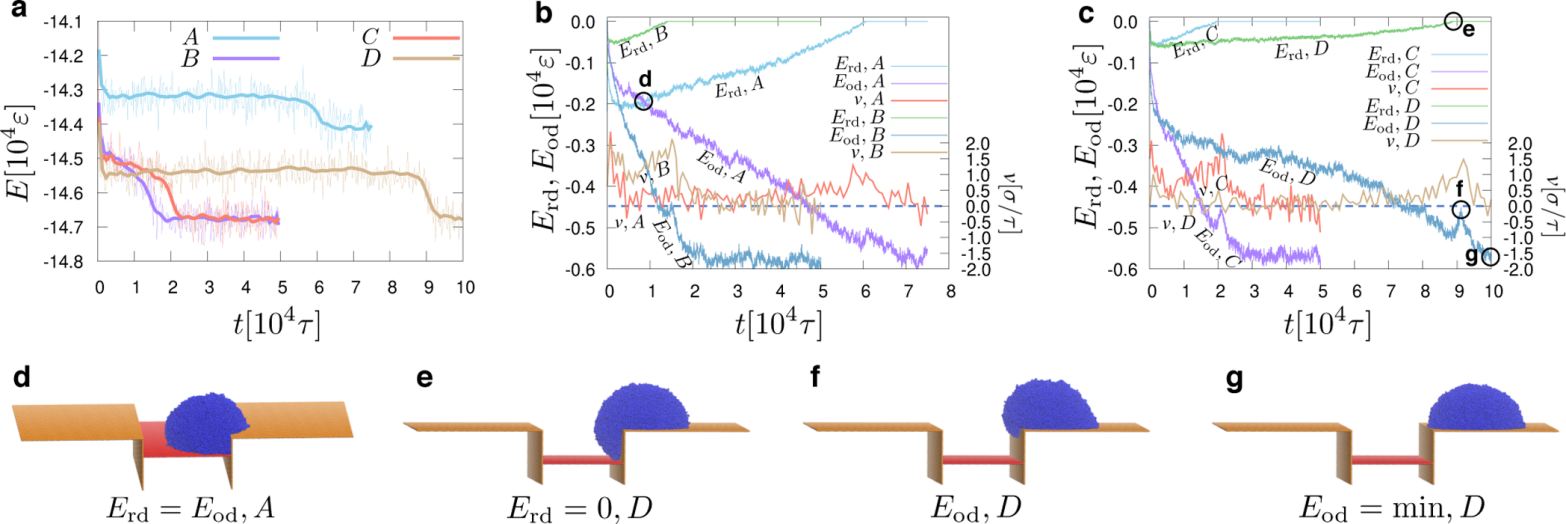
(a) Total potential energy, *E*, of four different systems (A, B, C, and D) based on a droplet with *N* = 50,400 beads and parameters, A: *H* = 12σ, ε_rd_ = 0.5ε, and ε_od_ = 0.7ε; B: *H* = 12σ, ε_rd_ = 0.3ε, and ε_od_ = 0.7ε; C: *H* = 16σ, ε_rd_ = 0.3ε, and ε_od_ = 0.7ε; and D: *H* = 21σ, ε_rd_ = 0.3ε, and ε_od_ = 0.7ε. Thick, solid lines are a guide for the eye. (b) Interfacial energy, *E*_rd_, between the red substrate and the droplet and the interfacial energy, *E*_od_, between the orange substrates and the droplet as a function of time, *t*, for systems A and B, as indicated. Also, the instantaneous velocity of the center of mass of the droplet in the *x* direction (*cf.*Figure 1), *v*, is plotted for systems A and B, as indicated. The dashed, horizontal line corresponds to *v* = 0.0σ/τ, and the corresponding values of the velocity are indicated on the right vertical axis of the graph. Negative values of *v* indicate that the droplet moves to the left, while positive values indicate that the droplet moves toward the positive direction of the *x* axis of our coordinate system. (c) Same as in panel (b), but the systems C and D are shown, as indicated. Lower panels (d–g) show snapshots at particular times for systems A and D, which are highlighted by a black circle on the graphs of panels (b,c) and indicated by the letter of the corresponding panel (d–g). The interfacial energies, *E*_rd_ and *E*_od_, which are relevant for our discussion in each case are shown below the snapshots for each case.

Figure 3 illustrates results that indicate whether droplets of different sizes (small, *N* = 1120 beads; medium, *N* = 10,080 beads; and large, *N* = 50,400 beads) can overcome a certain barrier of height *H*. It suggests that the cases with ε_rd_ = ε_od_ will always lead to pinned droplets irrespective of the droplet size. This is merely due to the physical pinning barrier, which, albeit small (*e.g.*, values as low as *H* = σ), is enough to hinder the beads attached to the substrate at the contact line to climb onto the orange substrate. Moreover, as discussed in the context of Figure 2, ε_od_ should always be larger than ε_rd_ for depinning to take place. In addition, the results of Figure 3 indicate it clearer that a larger difference between ε_od_ and ε_rd_ allows for the translocation of the droplet at higher values of *H*. A choice of ε_od_ as high as possible is desirable in order to favor depinning (also, for intermediate values of ε_rd_ and ε_od_), as suggested by Figure 3. Considering the case (ε_rd_ = 0.3ε, ε_od_ = 0.7ε), which enables barrier crossing for the highest values of *H* and clearly highlights the different areas in the graphs of Figure 4, we can see that low pinning barriers, *H* (*e.g.*, *H* < 8σ), will be overcome by all droplets, independently of their size. However, when *H* > 7σ, the medium and large droplets will only be able to cross the pinning barrier, *H*, while the small droplets will remain pinned. As *H* further increases, the medium-size droplets will remain pinned when *H* > 13, whereas the large droplets (*N* = 50,400 beads) will still be able to cross the pinning barrier. Finally, for *H* > 21, the large droplets will also remain pinned, being unable to overcome the pinning barrier. Hence, our results suggest that we can separate droplets of different sizes or control their motion in different directions by properly choosing the wettability of the red and the orange substrates (maximizing ε_od_ is desirable) and the height, *H*, of the pinning barrier. This approach could take place in multiple steps, where the small droplets will remain pinned at small *H*. Then, the medium-size droplets will remain pinned at higher *H* values and, finally, the larger values will remain pinned at higher *H*. Of course, different pinning barriers can be applied in different directions, thus implementing a binary code where certain droplets can either cross the pinning barrier or not. Our work clearly shows that the different behaviors are distinct and can be achieved by different choice of parameters.

In Figure 4, we provide details on the translocation mechanism of the droplet upon depinning as the droplet moves from the red substrate toward the top of the parallel orange substrate. For our discussion, we have selected four specific systems (see the caption of Figure 4), but our conclusions are valid for all of the successful depinning cases of Figures 2 and 3. The observed phenomena are dominated by the interfacial interactions; therefore, analysis of the different interfacial energy components, as well as the total energy of the system, should be carried out. In fact, the energy of the system provides the information for its most favorable state (toward equilibrium) for a particular set of parameters (*e.g.*, *H*, ε_rd_, ε_od_, and *N*) since the temperature remains constant throughout the simulation, while no changes in entropy are expected for the droplet and the substrate during the simulation. In particular, we show the pair potential interaction energy, *E*, for the selected systems, the interfacial energy between the droplet and the red substrate, *E*_rd_, as well as the interfacial energy between the droplet and the orange substrates, *E*_od_ (Figure 4). In fact, the latter interfacial contributions play the most important role in this translocation process. Indeed, these interfacial energies show significant deviations during the crossing of the pinning barrier (Figure 4b,c), which also arises from the wettability difference between the substrate. In particular, the ability of the droplet to establish more interactions (contacts between beads) with the orange substrates will eventually determine whether the droplet will be able to fully cross a pinning barrier of height *H*.

A closer look at the interfacial energies, *E*_rd_ and *E*_od_, provides more details on the mechanism of the barrier-crossing process (Figure 4b,c). During the crossing of the pinning barrier by the droplet, we observe that the energy *E*_rd_ gradually increases (its absolute value decreases, which means less contacts between the droplet beads and the beads of the red substrate). In contrast, *E*_od_ gradually decreases (faster decrease than the increase in *E*_rd_ also due to the fact that ε_rd_ < ε_od_), which manifests as an increasing number of contacts between the droplet and the orange substrates. At a specific time, for example, the one marked by the letter “d” in Figure 4b for system A and in the snapshot of Figure 4d, the two interfacial energies will be equal. In fact, *E*_rd_ and *E*_od_ will be equal for all systems at a certain time while crossing the pinning barrier. However, this happens very early in the depinning process when the difference between the parameters ε_rd_ and ε_od_ is large, as, for example, in the case of ε_rd_ = 0.3ε and ε_od_ = 0.7ε (systems B, C, and D). We underline that ε_od_ should always be larger than ε_rd_ in order for the droplet to be able to cross the pinning barrier, as seen, for example, from our results in Figure 2. On the contrary, when the wettability difference between the substrates is small (*e.g.*, in the case of system A, ε_rd_ = 0.5ε and ε_od_ = 0.7ε), *E*_rd_ = *E*_od_ at later times and when the droplet has considerably moved over the pinning barrier. In particular, when the parameters ε_rd_ and ε_od_ differ only by 0.1ε, then *E*_rd_ = *E*_od_ takes place when the droplet’s center of mass is halfway along the pinning barrier. Hence, the ability to choose the height of the pinning barrier, *H*, and the wettability of the red and orange substrates provides further possibilities for controlling the position of the droplet around the pinning barrier, in the cases where the droplet would remain pinned.

We now turn our attention to the dynamics of the droplet motion during the depinning process. At the initial stages of the barrier crossing, the instantaneous velocity of the center of mass of the droplet in the *x* direction, *v*, increases as the droplet seeks to establish more favorable contacts with the orange substrates (Figure 4b,c). However, as the droplet moves further along the pinning barrier, the competition between the red and the orange substrates to establish contacts with the droplet becomes higher since the droplet needs to climb up the pinning barrier in order to create new contacts with the top orange substrate. At this stage of the barrier crossing, the droplet moves back and forth and slowly drifts over the pinning barrier. After this stage and as the droplet moves further over the pinning barrier and because of the higher attraction of the droplet to the orange substrates (ε_od_ is always larger than ε_rd_), *E*_rd_ will become zero at some point in time and the droplet will lose its contact with the red substrate. For example, see point “e” in Figure 4c and the corresponding snapshot in Figure 4e for system *D*, which illustrates this effect. At this stage of the translocation process, the droplet is not dragged by the red substrate anymore and is “free” to establish further contacts with the top orange substrate. The absence of the attraction between the droplet and the red substrate leads to the increase of the instantaneous velocity, *v*, of the droplet, which, also, translates into the loss of some contacts with the orange substrate, as the droplet tries to obtain again its spherical-cap shape. This results in an increase of the energy, *E*_od_, which is marked in Figure 4c with the letter “f”. A snapshot that corresponds to this situation is presented in Figure 4f. Similar behavior has been discussed in the context of substrates with heterogeneity, where hysteresis builds up when the strength of the defect is above a certain threshold, which depends on the contributions of the elastic energy of the droplet and the barrier energy,^38^ which is strictly valid when gravitational effects are negligible.^39^ After this point, the droplet has managed to overcome the pinning barrier and climb on top of the orange substrate. However, the droplet has not yet completely reached its equilibrium shape. For example, the snapshot in Figure 4f clearly manifests this situation, since the advancing and receding contact angles of the droplet considerably differ. The droplet and generally the system as a whole will reach its final equilibrium state when it will establish a larger number of contacts with the orange substrate which is parallel to the *x* – *y*-plane. Then, as also *v* indicates, the droplet will move back and forth on the top substrate and will not return back to establish contacts with the perpendicular orange substrate or the red substrate. The number of interfacial contacts between the droplet and the substrate must always be maximized in order for the system to minimize its energy, which occurs only when the droplet eventually “sits” on the top substrate. Hence, the snapshot of Figure 4g (highlighted with the letter “g” in Figure 4c) is a typical equilibrium state of any system that can successfully overcome the pinning barrier. This conclusion is very important, for example, in a droplet separation process since it guarantees that the droplets that cross the pinning barrier will not return back to the red substrate. The description of the depinning mechanism, which we have provided here, is the same for all systems that cross the pinning barrier. However, for small *H*, the peak “f” of Figure 4 becomes less pronounced, as can be already hinted at by comparing the results for the systems of Figure 4. The same is true when the size of the droplet becomes smaller. Finally, we have mentioned that maximizing the wettability of the orange substrates (large value of the parameter ε_od_) is desirable in order to overcome ever higher pinning barriers (*cf.*Figures 2 and 3). We have concluded that the minimization of the interfacial energy, *E*_od_, is the driving force that enables the droplet to cross the pinning barrier.

**Figure 5:**
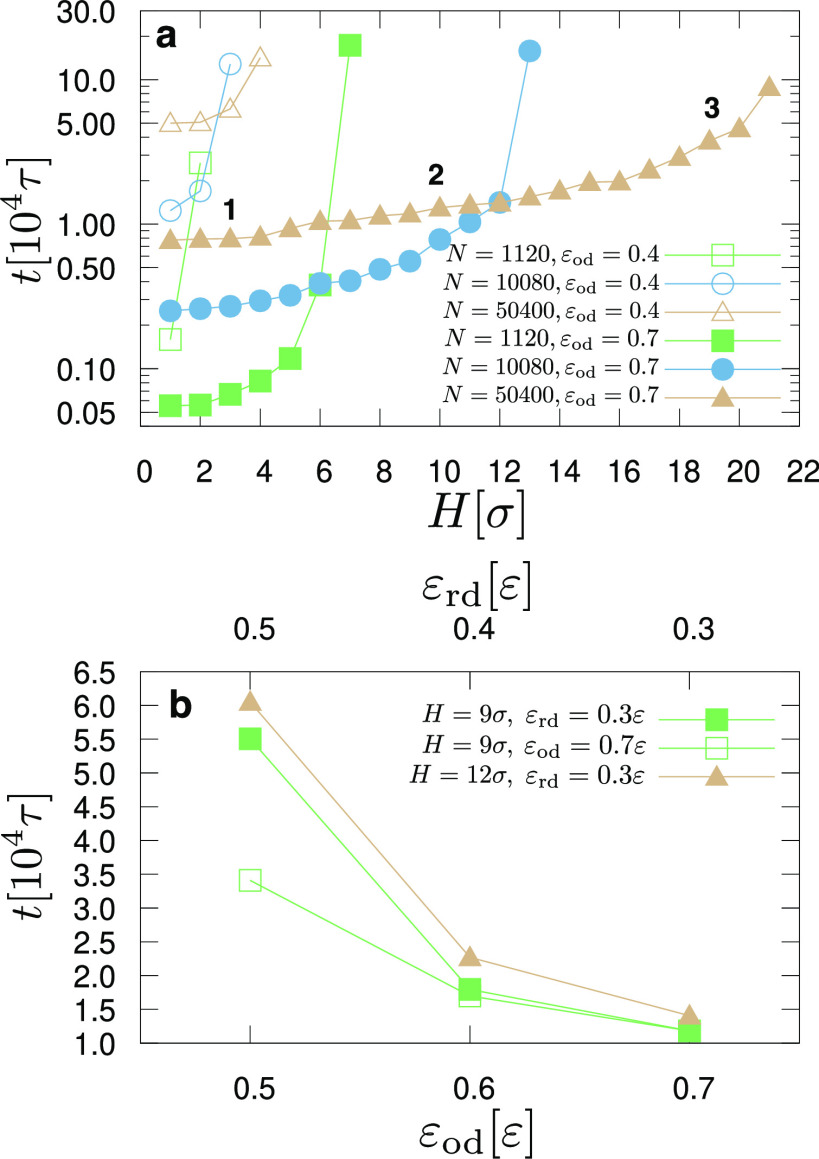
(a) Time required to cross the pinning barrier as a function of its height, *H*, for systems with ε_od_ = 0.4ε (open symbols) or ε_od_ = 0.7ε (filled symbols) for different droplet sizes (*N* = 1120: squares; *N* = 10,080: circles, and *N* = 50,400: triangles), as indicated. ε_rd_ = 0.3ε for all cases. (b) Time required to cross the pinning barrier as a function of ε_od_ (lower horizontal axis) when ε_rd_ = 0.3ε (filled symbols) or as a function of ε_rd_ (upper horizontal axis) when ε_od_ = 0.7ε (open symbols), as indicated. Cases of different heights, *H*, are shown, as indicated. The choice of these cases is based on the graphs of Figure 3, by choosing *H* values that allow the droplet to cross the pinning barrier for a range of values for the ε_rd_ and ε_od_ parameters.

Figure 5 presents results for the time required by the droplet to cross the pinning barrier. For the sake of our discussion, we show results of systems with very efficient barrier crossings, that is the difference in the wettability between the red and the orange substrates is maximized. Hence, ε_rd_ = 0.3ε and ε_od_ = 0.7ε. We contrast this behavior with the systems that exhibit the least efficient barrier crossings, that is, systems that can reach small *H*_max_ having a small difference in wettabilities, such as the choice ε_rd_ = 0.3ε and ε_od_ = 0.4ε. We have also considered different droplet sizes, as indicated in Figure 5a. Overall, all cases show that the time to cross the pinning barrier increases with the height *H*. While this dependence is monotonic, different behavior regimes can be observed. In particular, in the case of *N* = 50,400, ε_rd_ = 0.3ε, and ε_od_ = 0.7ε (Figure 5a), we can clearly discern three regimes. At the first regime (1, Figure 5a) for small values of *H*, namely σ < *H* < 5, the effect of the pinning barrier in the translocation process is very small due to the large size of the droplet. In this case, the increase of the barrier height, *H*, does not significantly affect the time that the droplets need to cross the pinning barrier. However, as the barrier, *H*, further increases, its effect on the time is more tangible, reflecting longer times that the droplet needs to cross the pinning barrier. This is the second regime (2, Figure 5a) characterized by an exponential growth in time. As we will see by comparison with the different cases of Figure 5, this exponent depends on both the size of the droplet and the particular choice of the parameters ε_rd_ and ε_od_. Hence, it is not possible to find a universal exponent for the crossing time, but we can observe that this exponent becomes smaller as the size of the droplet increases. The regime (1) may be a limiting case of this exponent when the effect of the pinning barrier becomes negligible on the time for the droplet to cross the barrier. In the third regime (3, Figure 5a), the droplet takes even more time to cross the pinning barrier and as *H* increases, this time practically becomes infinite. This behavior reflects the great difficulty of the droplet to further establish energetically favorable contacts with the top orange substrate. The above scenario for the largest droplet (*N* = 50,400 beads) also seems to apply in the case of smaller droplets (*i.e.*, *N* = 1120 and *N* = 10,080 beads) when the crossing is possible but with the exception that the behavior of regime (1) is absent. This simply means that values as low as *H* = σ already have an important influence on the translocation process in the case of the small droplets. This impact becomes even higher when the wettability difference between the red and the orange substrates is small (*e.g.*, ε_rd_ = 0.3ε and ε_od_ = 0.4ε, as shown in Figure 5a). In this case, some of the droplets are already exhibiting the behavior of regime (3), and the times to cross the pinning barrier increase by almost an order of magnitude for certain *H* values. Hence, we conclude that larger droplets offer better control in the time scale of the process when this is relevant for the application design. Our analysis is of course relevant in the absence of gravitational effects, that is length scales smaller than the capillary length, which is indeed the case in our *in silico* experiments.

Finally, we discuss how the time scale of the barrier crossing is affected by changes in the wettability between the substrates. Based on the results of Figures 2 and 3, we consider the cases shown in Figure 5b, for which we can observe barrier crossing for a wide range of parameters ε_rd_ and ε_od_ for fixed *H*. From the results of Figure 5b, we can conclude the following: first, choosing higher ε_od_ values leads to faster barrier crossings for the same difference between substrate wettability as expressed through the parameters ε_rd_ and ε_od_. Second, higher *H* values appear to affect proportionally the time of crossing the pinning barrier across the range of parameters ε_rd_ and ε_od_. Our conclusions seem to apply throughout the systems of this study. However, a more comprehensive discussion would still require larger droplets than the ones considered here, which goes beyond our current computational capabilities and the scope of this work.

## Conclusions

In this study, we have investigated the pinning of liquid droplets on solid substrates. We have discussed the necessary conditions for pinning and the mechanism of crossing the pinning barrier upon depinning. We found that even the smallest barrier, namely *H* = σ, is able to keep the droplet pinned when the wettability of the physical barrier is equal or smaller than the wettability of the substrate where the droplet “sits” before crossing the barrier. This is true for all droplet sizes considered in our study. Moreover, the crossing of a higher pinning barrier (*H*_max_) by the droplet is favored by the larger wettability of the substrates that form the barrier (orange substrates). In such cases, the crossing of the barrier will also be quicker. The time scale of the crossing depends on the size of the droplet, *N*, and the wettability of the substrates as expressed through ε_rd_ and ε_od_. In addition, we found that larger droplets can cross higher pinning barriers. We have analyzed in detail the mechanism of the barrier crossing and have identified the driving force of this process, which is the minimization of the system’s energy, with the main contribution coming from the decrease of the interfacial energy, *E*_od_, between the orange substrate and the droplet. To this end, we have presented a detailed discussion of the pinning–depinning mechanism and the barrier crossing by the droplet, and we have analyzed the dynamics of this process based on the instantaneous velocity of the center of mass of the droplet and the time scale of the crossing. For dynamics of movement, we have identified three different time scale regimes and discussed their implications for application exploitation. Furthermore, we have also described how pinning and depinning processes can be exploited in nanotechnology applications by controlling the droplet motion through a proper choice of the pinning barrier and the substrate wettabilities of the red and orange substrates for given droplet size. Our study provides ways of separating and steering droplets on solid substrates. In this way, we anticipate that our work could have direct implications in various nanotechology applications, especially in the areas of microfluidics, microfabrication, coatings, and biology.

## Supporting Information Available

Movies of barrier crossing by the droplets: M1*: *H* = 3.0σ, ε_rd_ = 0.3ε, ε_od_ = 0.5ε, *H* = 1120 beads; M2*: *H* = 10.0σ, ε_rd_ = 0.3ε, ε_od_ = 0.7ε, *H* = 10,080 beads; and M3*: *H* = 15.0σ, ε_rd_ = 0.3ε, ε_od_ = 0.6ε, *H* = 50,400 beads; and a movie of a pinned droplet (no barrier crossing) for the sake of comparison: M4*: *H* = 11.0σ, ε_rd_ = 0.3ε, ε_od_ = 0.4ε, *H* = 50,400 beads (ZIP)
